# Comprehensive analysis of lymphocyte subsets and transcriptome profiles in sepsis-induced acute respiratory distress syndrome: a prospective, observational study

**DOI:** 10.1016/j.clinsp.2025.100754

**Published:** 2025-08-22

**Authors:** Lei Yan, Zhimin Dong, Yumei Chen, Yi Han, Chaoyang Tong

**Affiliations:** Department of Emergency Medicine, Zhongshan Hospital, Fudan University, Shanghai, China

**Keywords:** Sepsis, Immune cell subpopulation, Acute respiratory distress syndrome, CD8^+^ T-cells

## Abstract

**Background:**

Inflammation caused by ongoing sepsis stimulation significantly contributes to immunosuppression. However, the alterations in lymphocyte subsets and transcriptome profiles in the development of sepsis-induced acute respiratory distress syndrome (ARDS) remain unclear.

**Methods:**

Peripheral blood mononuclear cells were collected from patients with sepsis at various points after admission. RNA sequencing was utilized to investigate the transcriptional changes in sepsis patients with and without ARDS. The immune cell composition of patients was dynamically monitored using flow cytometry. The comparisons were made between the changes in T cell subsets in sepsis patients without ARDS and those who developed ARDS during their hospitalization. Furthermore, the authors continuously tracked CD8^+^ T-cells in sepsis patients who progressed to ARDS. The role of CD8^+^ T-cells in the worsening of sepsis into ARDS was evaluated.

**Results:**

There were significant transcriptome gene differences between sepsis patients with and without ARDS. According to KEGG enrichment analysis, most of the differentially expressed genes between these patients were connected to infection, immunological response, cell proliferation, and death. Additional GO enrichment analysis revealed that many genes were linked to T-cell activation, proliferation, and growth. Compared with sepsis patients without ARDS, the number and percentage of lymphocytes, especially CD8^+^ T-cells, were lower in patients with sepsis-induced ARDS. The recovery of CD8^+^ T-cells in these patients was slow, too. The results of area analysis under the ROC curve suggested that by the seventh day of hospitalization, sepsis patients with ARDS typically have a CD8^+^ T-cell count of less than 162.5.

**Conclusion:**

Sepsis patients with and without ARDS exhibit markedly different genes and immune cells. Patients with ARDS typically exhibited reduced CD8^+^ T-cell counts and experienced a more protracted recovery process. Monitoring the immune cell composition of sepsis patients regularly and identifying ARDS as soon as possible is critical.

## Highlights

• Sepsis patients with and without ARDS exhibited significantly different gene expression and immune cell profiles.
• The number and proportion of lymphocytes, especially CD8^+^ T-cells, were lower in patients with sepsis-induced ARDS compared to those without ARDS. The recovery of CD8^+^ T-cells in these patients was also slow.
• By the seventh day of hospitalization, sepsis patients with ARDS generally had a CD8^+^ T-cell count of less than 162.5.

## Introduction

Immunoactivation and immunosuppression can occur sequentially or simultaneously in patients with sepsis.^1^, ^2^, ^3^, ^4^ During the early stages of sepsis, tissue damage or bacterial infection may trigger a rapid defense response, resulting in massive activation of immune cells and the widespread release of pro-inflammatory mediators.^3^ The immune system can restore balance if the pathogen is removed quickly.^3^^,^^5^ However, suppose the infection cannot be eradicated immediately. In that case, prolonged stimulation by antigens and inflammation may cause abnormalities in immune responses, potentially leading to immunological collapse and immunosuppression over time, a condition known as persistent inflammatory immunosuppressive catabolic syndrome.^4^^,^^6^^,^^7^ In other words, persistent and unresolved sepsis can alter innate and adaptive immune responses through immunosuppression, accompanied by ongoing inflammation, resulting in recurrent and secondary infections.^1^^,^^4^ Most individuals with sepsis survive the initial storm of inflammation. However, there is increasing evidence that a prolonged period of immunosuppression may contribute to nosocomial infections secondary to sepsis and accelerate multiple organ dysfunction.^2^ Therefore, many scholars believe that immunosuppression related to inflammation is a critical factor in the poor prognosis of sepsis patients.^7^^,^^8^

T-cell count is essential for predicting sepsis-induced immunosuppression.^4^^,^^15^ Sustained antigenic and inflammatory stimulation in sepsis results in a significant reduction in the number and efficacy of T-cells due to apoptosis and high expression of suppressive immune checkpoint molecules (PD-1, Tim-3), which promotes immunosuppression and even leads to immune system collapse.^11^, ^12^, ^13^ The changes in T-cell subsets in sepsis patients have significant clinical predictive value: the reduction of the CD4^+^/CD8^+^ T-cell ratio in trauma patients directly correlates with the risk of sepsis and multiple organ dysfunction syndrome.^8^ Therefore, comparing the transcriptome genes and the subtypes of T-cells in blood is clinically important for a dynamic understanding of sepsis-associated organ dysfunction.

The previous study discovered reduced CD8^+^ T-cell expression in the blood of patients with sepsis-related acute respiratory distress syndrome (ARDS) .^15^^,^^16^ The host's capacity to phagocytose and eliminate pathogens is compromised when CD8^+^ T-cells are diminished.^17^^,^^18^ Further impairment of the patient's immune defense system may promote multiple organ dysfunction.^19^ However, the role of T-cell subsets, particularly CD8^+^ T-cells, in developing sepsis-induced ARDS has not been thoroughly studied. Therefore, this study will analyze lymphocyte changes and transcriptomes in peripheral blood mononuclear cells from patients with sepsis alone and those with secondary ARDS. This study aimed to evaluate the predictive value of T-cell subsets and transcriptome profiles in progressing sepsis to ARDS.

## Methods

### Study design and patients’ enrollment

In this study, patients with sepsis were recruited between December 2018 and March 2022 for a prospective observational study. Patients diagnosed with sepsis during hospitalization (within 24 h) in the emergency department were included in this study according to the 2016 “Surviving Sepsis Campaign” guidelines.^14^^,^^20^ According to the Berlin diagnostic criteria, ARDS was defined by the following parameters: (1) Acute onset of bilateral infiltrates on chest radiograph or CT; (2) Mechanical ventilation and positive end-expiratory pressure or continuous positive airway pressure ≥5 cmH_2_O; (3) Severe (PaO_2_/FiO_2_ ≤ 100 mmHg), moderate (PaO_2_/FiO_2_ = 100–200 mmHg), or mild (PaO_2_/FiO_2_ = 200–300 mmHg); (4) Without pleural effusion, lung collapse, lung nodules, or cardiogenic pulmonary edema.^21^ At enrollment, all patients fulfilled the diagnostic criteria for sepsis but not ARDS. Informed consent was obtained from all patients or their legal representatives before enrollment. This study was conducted in the emergency department of the studied hospital, and the study was approved by the hospital ethics committee. This study followed the STROBE Statement.

The inclusion criteria were as follows: (1) Greater than 18 and less than 80-years-old, and gender is not limited; (2) Body Mass Index (BMI): 18.1∼27.9 kg/m^2^; (3) Met the diagnostic criteria for sepsis. Patients were excluded if any of the following criteria were fulfilled: younger than 18 or older than 80, end-stage chronic disease, and estimated survival time < 28 days, autoimmune disease, immunodeficiency, tumor, chronic infectious diseases, received chemotherapy within 6-months, or consent could not be obtained.

The study was approved by the ethics committee of Zhongshan Hospital, affiliated with Fudan University (B2021-596R). Written informed consent was obtained from patients or their guardians.

### Data collection

For patients diagnosed with sepsis, peripheral blood samples were collected upon admission (D0), on the 7th day post-admission (D7), and the 14th day post-admission for further analysis (D14). The following information on each patient was extracted from electronic medical records: demographic data, the site of infection, organ function, cytokine, disease severity, prescribed drugs, mechanical ventilation parameters, and prognosis. Acute Physiology and Chronic Health Evaluation II (APACHEII) scores, Sequential Organ Failure Assessment (SOFA) scores, and Lung Injury Prediction Score (LIPS) scores were assessed by the supervising physician based on the daily changes in the patient's condition.^9^^,^^10^^,^^30^ Patients were categorized into Sepsis alone and Sepsis + ARDS groups based on whether they progressed to ARDS within 14-days.^20^, ^21^, ^22^, ^23^

### Isolation of peripheral blood mononuclear cells (PBMCs)

Peripheral blood samples were diluted 1:1 with phosphate-buffered saline before the separation of PBMCs by density gradient centrifugation. Cells were washed in RPMI1640 supplemented with 10 % fetal calf serum and used immediately.

### RNA extraction and RNA-sequencing

Total RNA was extracted from sorted human PBMC using TRIzol reagent (Thermo Fisher Scientific, Massachusetts, USA) according to the manufacturer’s instructions. Subsequently, total RNA was qualified and quantified using a NanoDrop and Agilent 2100 Bioanalyzer (Thermo Fisher Scientific). The mRNA was purified using oligo (dT)-attached magnetic beads for mRNA library construction, and the purified mRNA was fragmented into small pieces with the fragment buffer at the appropriate temperature. First-strand cDNA was generated using random hexamer-primed reverse transcription, followed by second-strand cDNA synthesis. Then, A-Tailing Mix and End Repair Mix were added to repair the ends. The cDNA fragments obtained from the previous step were acquired and amplified by PCR. RNA libraries were prepared for sequencing using standard Illumina protocols. RNA sequencing was performed on an Illumina Novaseq 6000 platform using 150-bp single-end reads. Sequencing of the same sample in two lanes showed comparable results.

### Flow cytometry

CD3^+^ /CD4^+^ /CD8^+^ T-cell, CD19^+^ B-cell, and CD56^+^ NK-cell counts (cells/μL) were measured by multiple-color flow cytometry with human monoclonal anti-CD3-FITC, anti-CD4-PE-cy7, anti-CD8-APC-cy7, anti-CD19-APC, anti-CD56-PE antibodies (BD Multitest) according to the manufacturer’s instructions. The cells were analyzed on a BD FACS Canto II flow cytometry system (BD Biosciences).

## Statistical analysis

Data were entered and analyzed using Statistical Package for Social Sciences version 22.0 (IBM, USA, New York) and GraphPad Prism version 8.0 (GraphPad Software, California, United States of America). Group comparisons were performed to compare the differences in T-cell subtypes across groups (with and without ARDS). Normally distributed data were reported with means and standard deviations and analyzed using independent samples *t*-tests. Non-normally distributed data were reported with medians and interquartile ranges and were analyzed with a Mann-Whitney *U* test. Categorical data were compared using the chi-square or Fisher’s exact test. The area under the Receiver Operating Characteristic (ROC) curve was calculated to evaluate the prognostic value of the tested parameters; *p* ≤ 0.05 was considered statistically significant.

## Results

### Clinical and laboratory characteristics

**Fig. 1:**
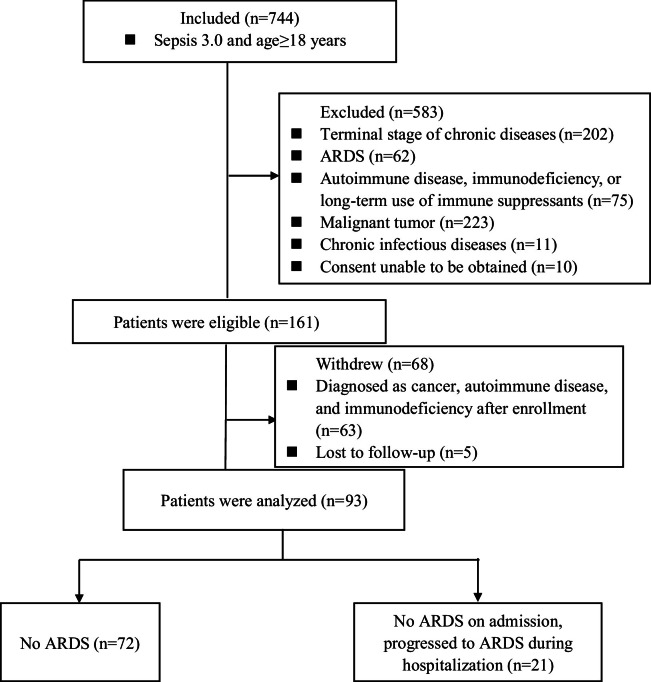
Flow of patient screening and enrollment.

A total of 744 patients with sepsis were prospectively enrolled in this study. Of these, 583 patients were excluded based on exclusion criteria, and 68 were withdrawn from the study. Ultimately, 93 patients completed the research. During the observational follow-up, 72 patients did not develop ARDS, while 21 developed it within two weeks of admission. Consequently, the patients were divided into two groups: those with sepsis alone, who did not develop ARDS (72), and those with sepsis-induced ARDS (21) (Fig. 1).

The clinical characteristics of all patients at admission are shown in Table 1. Compared to patients with sepsis alone, those who developed ARDS throughout their illness had greater APACHEII, SOFA, and LIPS scores (*p* < 0.05). At admission, this group of patients progressing to ARDS had a lower oxygenation index than the control group (*p* < 0.05). There was no significant difference between the two groups of patients in terms of other clinical data (age, gender, main infection foci, comorbidities, cytokines, and so on).

**Table 1:** Baseline characteristics of the patients enrolled in the study.

	Sepsis without ARDS (*n* = 72)	Sepsis-induced ARDS (*n* = 21)	p-value
Baseline demographic			
Age, years	62.93 ± 14.58	65.81 ± 13.44	0.42
Male/Female, n	43/29	17/4	0.07
BMI, kg/m^2^	24.28 ± 4.79	25.03 ± 3.68	0.65
Primary infection, n ( %)			
Pneumonia	22 (30.56)	8 (38.10)	0.52
Abdominal infection	41 (56.94)	12 (57.14)	0.99
Infective endocarditis	4 (5.56)	0 (0)	0.13
Bloodstream infection	3 (4.17)	1 (4.76)	1
Others	2 (2.78)	0 (0)	1
Comorbidities, n ( %)			
Diabetes mellitus	29 (40.28)	5 (23.81)	0.17
COPD	4 (5.56)	2 (9.52)	0.88
Renal failure	25 (34.72)	11 (52.38)	0.14
Disease severity			
APACHE II Score	12.01 ± 7.00	18.33 ± 8.89	0.001
SOFA Score	4 (2,7)	6 (4,11)	0.006
LIPS Score	3.5 (2,5.5)	6.5 (4.25, 8.25)	0.001
PaO_2_/FiO_2_	268.33 ± 114.11	190.97 ± 76.09	0.004
Cytokine (pg/mL)			
TNF-α	26.30 (17.78, 43.83)	24.30 (12.55, 51.15)	0.626
IL-1β	8.50 (5.00, 14.25)	8.90 (5.15, 17.60)	0.399
IL-2R	1346.50 (993.25, 2905.00)	1336.00 (642,3359)	0.476
IL-6	8.50 (5.00, 14.25)	8.90 (5.15, 17.60)	0.399
In-hospital mortality, n ( %)	1 (1.39)	10 (47.62)	<0.001
Mechanical ventilation, n ( %)	9 (12.50)	6 (28.57)	0.15
Prescribed drugs, n ( %)			
Vasopressors	17 (23.61)	5 (23.81)	1
Glucocorticoid	9 (12.50)	8 (38.10)	0.019

### RNA transcriptome analysis in sepsis patients with or without ARDS

In this study, the authors first performed gene transcriptome analysis of PBMC-extracted RNA from eight sepsis patients with or without ARDS. The authors first screened the Differentially Expressed Genes (DEGs) in the two groups of patients. The authors applied the ‘limma’ *R* package to identify DEGs and set *p* < 0.05 and [logFC] ≥ 1 as the cut-off value (Supplementary Material 1A‒B). In total, 1821 common DEGs were identified between these two groups of patients, consisting of 1035 upregulated genes and 786 downregulated genes. Bioinformatic analysis was used to further annotate the identified DEGs, including KEGG signaling pathway enrichment and GO terms enrichment. According to KEGG enrichment analysis, most of the differentially expressed genes between patients with simple sepsis and patients with ARDS in this study were connected to infection, immunological response, cell proliferation, and death (Supplementary Material 1E). Additional GO enrichment analysis revealed that many genes were linked to T-cell activation, selection, receptor signaling pathway, proliferation, and differentiation (Supplementary Material 1C‒D).

### Changes in lymphocyte subsets of sepsis patients with or without ARDS

**Fig. 2:**
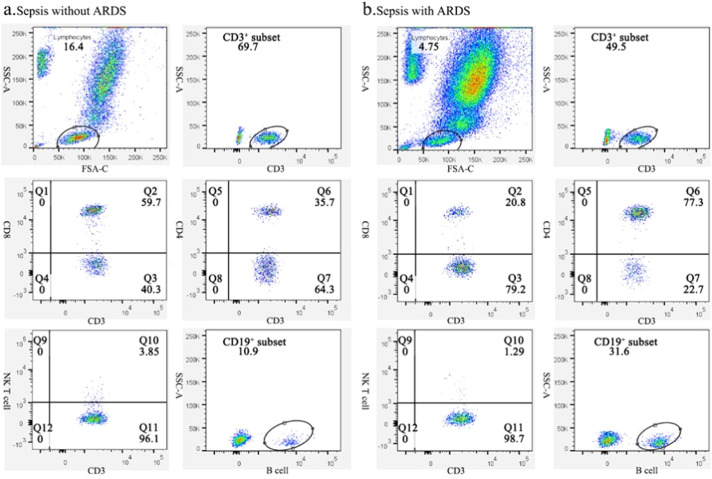
Representative flow charts of lymphocyte subpopulations in sepsis patients with and without ARDS. (A) Representative flow plots of lymphocytes, CD3^+^ T-cells, CD8^+^CD3^+^ T-cells, CD4^+^CD3^+^ T-cells, NK cells, and B-cell subsets in sepsis patients without ARDS. (B) Representative flow diagrams of lymphocyte, CD3^+^ T-cells, CD8^+^CD3^+^ T-cells, CD4^+^CD3^+^ T-cells, NK cells, and B-cells subsets in patients who deteriorated into ARDS.

Based on the gene transcriptome analysis results, the authors focused on changes in lymphocyte subsets in both groups. By flow cytometry, the authors examined each subpopulation of peripheral blood lymphocytes at days 0 and 7 in both groups. Fig. 2 depicts a typical flow chart for each cell subset in two patients, one with sepsis without ARDS and the other with sepsis-induced ARDS. The proportion and amount of T-cells, CD8^+^ T*-*cells, B-cells, and NK cells in patients with simple sepsis and those with subsequent ARDS are shown in Table 2.

**Table 2:** Changes of the subsets of lymphocytes in sepsis patients with and without ARDS.

Lymphocyte subset	D0			D7		
	Without ARDS	With ARDS	p-value	Without ARDS	With ARDS	p-value
Lymphocytes (10^9^/L)	0.90 ± 0.51	1.29 ± 1.64	0.08	1.25 ± 0.65	0.89 ± 0.75	<0.05
B-cells ( %)	22.18 ± 11.35	22.87 ± 10.01	0.80	13.36 ± 7.79	25.06 ± 14.54	<0.01
B-cells (cells/uL)	181.92 ± 127.46	200.81 ± 187.50	0.60	163.57 ± 122.48	106.00 (55.00, 198.50)	0.49
T-cells ( %)	63.08 ± 12.00	59.78 ± 12.77	0.28	71.77 ± 9.55	57.15 ± 18.77	<0.01
T-cells (cells/uL)	566.14 ± 377.17	551.10 ± 438.70	0.88	924.38 ± 529.17	411.86± 278.64	<0.01
CD8^+^ T-cells ( %)	21.83 ± 9.87	18.04 ± 9.21	0.12	26.26 ± 9.97	18.89 ± 9.61	<0.05
CD8^+^ T-cells (cells/uL)	194.19 ± 143.71	150.43 ± 120.88	0.21	268.00 (182.50, 420.50)	132.52 ± 112.18	<0.01
NK cells ( %)	13.50 ± 8.12	16.03 ± 9.96	0.24	13.93 ± 8.24	16.67 ± 9.48	0.20
NK cells (cells/uL)	113.69 ± 92.89	120.76 ± 127.88	0.78	122.00 (91.25, 233.75)	112.10 ± 89.76	<0.05
WBCs (10^9^/L)	13.12 ± 7.79	11.79 ± 6.56	0.48	9.18 ± 5.13	11.40 ± 6.47	0.16
Neutrophil-to-lymphocyte ratio	12.64 (7.19, 23.28)	15.32 ± 11.86	0.73	6.72 ± 4.86	18.68 ± 20.31	0.01

Although there was no significant difference in white blood cell counts between the two groups on days 0 and 7, ARDS patients exhibited a significantly lower neutrophil-to-lymphocyte ratio (*p* = 0.01). There was no significant change in B-cell numbers on day 0 and day 7, regardless of whether patients progressed to ARDS. Additionally, no significant variation was identified in the proportion of NK cells between the two groups. On the seventh day of hospitalization, T-lymphocyte and CD8^+^ T-cell counts in ARDS patients were significantly lower than in sepsis patients (Table 2).

### Changes of CD8^+^ T-cells in sepsis patients with and without ARDS

**Fig. 3:**
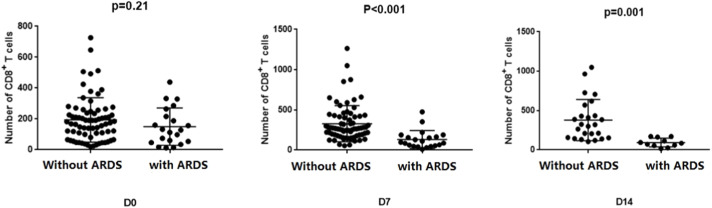
Differences in CD8^+^ T cell counts between the two groups at various time points. Comparison of peripheral blood CD8^+^ T-cell counts on day 0, day 7, and day 14 in sepsis patients with and without ARDS.

The CD8^+^ T-cell counts of the two groups were compared and analyzed on days 0, 7, and 14 (Fig. 3). At admission, there was no statistically significant difference in CD8^+^ T-cell counts between the two groups (*p* = 0.21). However, sepsis patients without ARDS exhibited significantly higher CD8^+^T-cell counts than those with ARDS after one week (*p* < 0.001). This difference in variance remained significant after two weeks (*p* = 0.001).

### Comparison of the number of CD8^+^ T-cells between the two groups

**Fig. 4:**
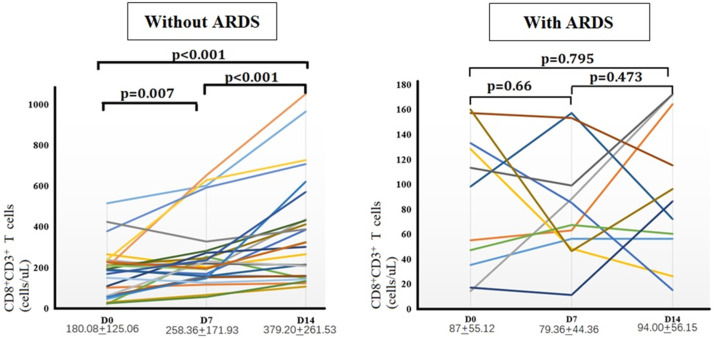
Dynamics of CD8^+^ T-cells in the two cohorts. Differences in peripheral blood CD8^+^ T-cell counts between sepsis patients with and without ARDS at various points (days 0, 7, and 14).

Next, the authors further monitored peripheral CD8^+^ T-cells dynamically in 25 patients with sepsis without ARDS and 11 patients with sepsis-induced ARDS on days 0, 7, and 14 (Fig. 4). Sepsis patients without ARDS experienced a significant increase in CD8^+^ T-cells in peripheral blood over time. The number of CD8^+^ T-cells on day 14 was significantly greater than on days 0 and 7 (*p* < 0.05). In contrast, peripheral CD8^+^ T-cell counts in patients with sepsis-induced ARDS remained continuously low during the first two weeks, with no statistically significant differences observed at any of the three time points.

### Predictive role of CD8^+^ T-cells for secondary ARDS in sepsis patients

**Fig. 5:**
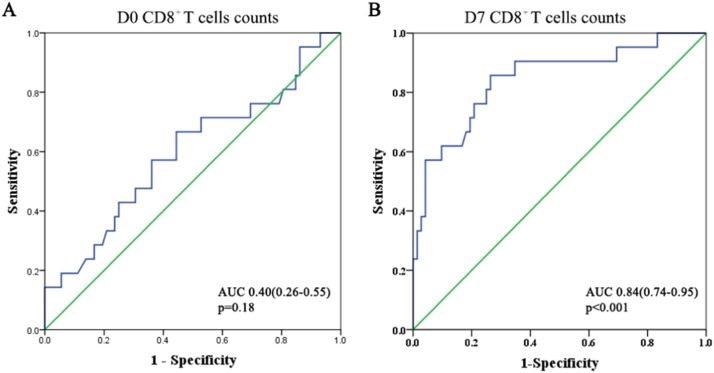
ROC curve of peripheral CD8^+^ T-cells for predicting secondary ARDS in sepsis patients.

Using the ROC curve, the authors evaluated the predictive value of CD8^+^ T-cell counts for secondary ARDS in patients with sepsis (Fig. 5). At admission, the area under the ROC curve for CD8^+^ T-cells was approximately 0.4, indicating a poor prognostic value for the development of ARDS (*p* = 0.18). However, this area increased to 0.84 after one week. The Youden's index reached its optimal value when the CD8^+^ T-cell count was 162.5 on day 7. This indicates that by the seventh day of hospitalization, patients with sepsis-induced ARDS often have a CD8^+^ T-cell count below 162.5. When the CD8^+^ T*-*cell count in sepsis patients drops below 162.5, it is essential to monitor for sepsis-induced ARDS.

## Discussion

ARDS often occurs in patients due to various factors that promote a systemic inflammatory response, including sepsis, near-drowning, severe trauma, or multiple blood transfusions, with sepsis being the most common cause of ARDS.^24^, ^25^, ^26^ In this study, sepsis patients with secondary ARDS exhibited higher APACHEII and SOFA scores than those without ARDS on day 0. This indicated that the immune system and physiological balance were more adversely affected by the inflammatory damage caused by the septic cytokine storm, resulting in more severe organ damage. Consequently, the mortality rate for these patients was significantly higher than for those without ARDS.

The pathogen infection rapidly heightens the body's immune response during the acute phase of sepsis, leading to excessive activation of immune cells and substantial production of pro-inflammatory mediators.^2^^,^^27^^,^^28^ If the infection is promptly controlled, the immune system can be quickly restored to balance.^5^ However, if the pathogenic germs are not swiftly eradicated, persistent antigenic stimulation and damage may result in impaired proliferation and dysfunction of immune cells.^7^^,^^29^, ^30^, ^31^, ^32^ Patients in this situation are at risk for secondary infections, which may lead to long-term immunosuppression or collapse, resulting in multi-organ failure.^33^ In sepsis-induced multiple organ dysfunction, the lung is typically the most frequently implicated organ.^3^^,^^15^^,^^16^

This research analyzed gene expression in PBMCs from sepsis patients with secondary ARDS and those without ARDS. Significant differences in transcriptome genes were observed between sepsis patients with ARDS and those without. GO enrichment analysis indicated that the functional enrichment of DEGs primarily involved lymphocyte proliferation, regulation of T-cell proliferation, activation and differentiation, the T-cell receptor signaling pathway, and the establishment and translation of protein localization to the membrane. This suggests that cell membrane proteins may participate in T-cell-mediated immunity and the pathological process of sepsis-induced ARDS. T-cell proliferation, differentiation, and functional changes could significantly contribute to the exacerbation of sepsis to ARDS. KEGG analysis revealed that the enrichment of DEGs was mainly related to infectious diseases (viruses, bacteria, and parasites), the immune system, cell growth and death, the cellular community of eukaryotes, signaling molecules, and signal transduction. It is suggested that lymphocyte dysfunction, particularly T-cell dysfunction, may be a critical factor in respiratory dysfunction secondary to sepsis. Consistent with other findings, immunocyte dysfunction regulated by T-cells may play an integral role in sepsis and its related complications.^15^^,^^28^^,^^34^^,^^35^

The hypothesis was further tested by analyzing the cellular immune composition of sepsis patients, specifically focusing on changes in lymphocyte subsets among those who developed ARDS. At admission, no significant difference was observed in the number of CD8^+^ T-cells between sepsis patients with and without ARDS. Despite receiving equally aggressive treatment, CD8^+^ T-cells increased rapidly in sepsis patients without ARDS over the course of one week, and these levels were significantly higher than in patients who progressed to ARDS, with this difference remaining evident after two weeks. Individual analysis of each group showed that peripheral CD8^+^ T-cells in sepsis patients without ARDS demonstrated an increasing trend over time, whereas in patients with sepsis-induced ARDS, peripheral CD8^+^ T-cells remained at a lower level throughout the illness. The study found that lymphocytes, especially CD8^+^ T-cells, were present in lower numbers and percentages among sepsis-induced ARDS patients for at least two weeks. This indicated that the CD8^+^ T-cells in these patients had limited capacity for growth and activity, reducing their ability to fully contribute to the inflammatory response and pathogen elimination in sepsis.

The area under the ROC curve analysis in this study revealed that sepsis patients were more likely to progress to ARDS if their CD8^+^ T-cell counts remained below 162.5 after one week of vigorous therapy. This study encourages emergency physicians to pay special attention to immune cell composition in the clinical diagnosis and management of sepsis patients, as these patients are more likely to develop ARDS if their CD8^+^ T-cells are low and slow to recover. When CD8^+^ T-cells are reduced and recovery is sluggish, these patients are at risk of developing ARDS. Therefore, early prevention and treatment strategies, such as mechanical ventilation, prone ventilation, and volume modification, are necessary for these sepsis patients.^1^

Sepsis-induced ARDS leads to a decrease in CD8^+^ T-cell numbers and functionality. The previous study indicated that CD8^+^ T-cell differentiation and proliferation can vary among patients with sepsis-induced ARDS.^15^ Further research shows that CD8^+^ T-cells displayed signs of exhaustion in sepsis-induced ARDS mouse models. This phenotype may result from ongoing antigenic or inflammatory stimulation, in addition to the ischemic and hypoxic microenvironment, which might impair the proliferation and function of immune cells.^36^ However, the group is still exploring the regulation and mechanisms governing the proliferation, differentiation, and function of CD8^+^ T-cells in patients with sepsis-induced ARDS. Nonetheless, the present study had several limitations. Firstly, this investigation was limited to a single medical institution, and the number of cases was too low to eliminate the possibility of statistical data bias affecting the findings. Secondly, this study concentrated on CD8^+^ T-cells, and examining other subpopulations would strengthen the credibility of the results. It is worth noting that single-cell RNA sequencing could offer additional insights into this research. Nevertheless, this study provides new perspectives on the molecular mechanisms and immunopathology of sepsis-related ARDS.

## Conclusion

This study discovered that CD8^+^ T-cell counts were consistently low and that recovery was delayed in patients who progressed to ARDS. This implies that the immunological functions mediated by CD8^+^ T-cells, such as killing pathogens and lysing target cells, may be deficient in patients with sepsis-induced ARDS. However, a low number of CD8^+^ T-cells does not necessarily indicate functional defects. It remains to be verified what changes occur in CD8^+^ T-cells and how they affect prognosis. Therefore, the authors need to examine further the proliferation, surface molecular expression, and cytokine secretion of CD8^+^ T-cells in future studies.

## Abbreviations

APACHE, Acute Physiology and Chronic Evaluation; ARDS, Acute Respiratory Distress Syndrome; BMI, Body Mass Index; DEGs, Differentially Expressed Genes; LIPS, Lung Injury Prediction Score; PBMCs, Peripheral Blood Mononuclear Cells; ROC, Receiver Operating Characteristics; SOFA, Sequential Organ Failure Assessment.

## Availability of data and materials

The datasets generated and/or analyzed during the current study are not publicly available due to privacy and ethical restrictions, but they are available from the corresponding author upon reasonable request.

## Consent for publication

Not Applicable.

## Ethics approval and consent to participate

This study obtained ethics approval from the Ethics Committee of Zhongshan Hospital affiliated to Fudan University. The approval number is B2021-596R. The written informed consent was obtained from the patient or the patient’s guardian. All of the authors of this study confirmed that all methods were carried out in accordance with relevant guidelines and regulations (Declaration of Helsinki).

## Authors' contributions

L. Yan and CY. Tong designed the study. ZM. Dong performed the research and collected the data. YM. Chen and Y. Han carried out the flow cytometric analysis. L. Yan analyzed the data and wrote the manuscript. All authors read and approved the final version of the manuscript.

## Funding

This study was funded by grants from the Young Fund of Zhongshan Hospital (2018ZSQN47) and 10.13039/501100014220China National Natural Science Fund (82102255).

## Declaration of competing interest

The authors declare no conflicts of interest.

## Appendix Supplementary materials

Image, application 1

## References

[1] Liu D., Huang S.-Y., Sun J.-H., Zhang H.-C., Cai Q.-L., Gao C. (2022). Sepsis-induced immunosuppression: mechanisms, diagnosis and current treatment options. Mil Med Res.

[2] Yao R.-Q., Ren C., Zheng L-Y, Xia Z.-F., Yao Y.-M. (2022). Advances in Immune Monitoring Approaches for Sepsis-Induced Immunosuppression. Front Immunol..

[3] Kumar V. (2020). Pulmonary Innate Immune Response Determines the Outcome of Inflammation During Pneumonia and Sepsis-Associated Acute Lung Injury. Front Immunol.

[4] Nakamori Y., Park E.J., Shimaoka M. (2020). Immune Deregulation in Sepsis and Septic Shock: reversing Immune Paralysis by Targeting PD-1/PD-L1 Pathway. Front Immunol.

[5] van der Poll T., Van De Veerdonk F.L., Scicluna B.P., Netea Mihai G (2017). The immunopathology of sepsis and potential therapeutic targets. Nat Rev Immunol.

[6] Rosenthal M.D., Moore F.A. (2016). Persistent Inflammation, Immunosuppression, and Catabolism: evolution of Multiple Organ Dysfunction. Surg Infect.

[7] Fenner B.P., Darden D.B., Kelly L.S., Rincon J., Brakenridge S.C., Larson S.D. (2020). Immunological Endotyping of Chronic Critical Illness After Severe Sepsis. Front Med (Lausanne).

[8] Menges T., Engel J., Welters I., Wagner R.M., Little S.R., Ruwoldt R. (1999). Changes in blood lymphocyte populations after multiple trauma: association with posttraumatic complications. Crit Care Med.

[9] Ferreira F.L., Bota D.P., Bross A., Mélot C., Vincent J.L. (2001). Serial evaluation of the SOFA score to predict outcome in critically ill patients. JAMA.

[10] Zhao F., Shen Z., Yang C., Cong Z., Zhang H., Zhu X. (2022). Establishment and verification of LIPS score combined with APACHEII score and oxygenation index to predict the occurrence model of ARDS. Zhonghua Wei Zhong Bing Ji Jiu Yi Xue.

[11] Padovani C.M., Yin K. (2024). Immunosuppression in Sepsis: biomarkers and Specialized Pro-Resolving Mediators. Biomedicines.

[12] Venet F., Monneret G. (2018). Advances in the understanding and treatment of sepsis-induced immunosuppression. Nat Rev Nephrol.

[13] Hudson W.H., Gensheimer J., Hashimoto M., Wieland A., Valanparambil R.M., Li P. (2019). Proliferating Transitory T-cells with an Effector-like Transcriptional Signature Emerge from PD-1(+) Stem-like CD8(+) T-cells during Chronic Infection. Immunity.

[14] Dellinger R.P., Levy M.M., Rhodes A., Annane D., Gerlach H., Opal S.M. (2013). Surviving Sepsis Campaign Guidelines Committee including The Pediatric Subgroup. Surviving Sepsis Campaign: international guidelines for management of severe sepsis and septic shock 2012. Intensive Care Med.

[15] Yan L., Chen Y., Han Y., Tong C. (2022). Role of CD8(+) T-cell exhaustion in the progression and prognosis of acute respiratory distress syndrome induced by sepsis: a prospective observational study. BMC Emerg Med.

[16] Yan L., Tong C.Y. (2023). Analysis of blood immune cell and gene microarray data of patients with sepsis related Acute Respiratory Distress Syndrome based on GEO database. Fudan Univ J Med Sci.

[17] Belk J.A., Daniel B., Satpathy A.T. (2022). Epigenetic regulation of T-cell exhaustion. Nat Immunol.

[18] Barili V., Vecchi A., Rossi M., Montali I., Tiezzi C., Penna A. (2021). Unraveling the Multifaceted Nature of CD8 T-Cell Exhaustion Provides the Molecular Basis for Therapeutic T-cell Reconstitution in Chronic Hepatitis B and C. Cells.

[19] Cao M., Wang G., Xie J. (2023). Immune dysregulation in sepsis: experiences, lessons and perspectives. Cell Death Discov.

[20] Singer M., Deutschman C.S., Seymour C.W., Shankar-Hari M., Annane D., Bauer M. (2016). The Third International Consensus Definitions for Sepsis and Septic Shock (Sepsis-3). JAMA.

[21] Definition Task Force A.R.D.S., Ranieri V.M., Rubenfeld G.D., Thompson B.T., Ferguson N.D., Caldwell E., Fan E. (2012). Acute respiratory distress syndrome: the Berlin Definition. JAMA.

[22] Kempker J.A., Martin G.S. (2016). The Changing Epidemiology and Definitions of Sepsis. Clin Chest Med.

[23] Matthay M.A., Zemans R.L. (2011). The acute respiratory distress syndrome: pathogenesis and treatment. Annu Rev Pathol.

[24] Li S., Zhao D., Cui J., Wang L., Ma X., Li Y. (2020). Prevalence, potential risk factors and mortality rates of acute respiratory distress syndrome in Chinese patients with sepsis. J Int Med Res.

[25] Abe T., Madotto F., Pham T., Nagata I., Uchida M., Tamiya N. (2018). LUNG-SAFE Investigators and the ESICM Trials Group. Epidemiology and patterns of tracheostomy practice in patients with acute respiratory distress syndrome in ICUs across 50 countries. Crit Care.

[26] Hu Q., Hao C., Tang S. (2020). From sepsis to acute respiratory distress syndrome (ARDS): emerging preventive strategies based on molecular and genetic researches. Bioscience Rep.

[27] Nedeva C., Menassa J., Sepsis Puthalakath H. (2019). Inflammation Is a Necessary Evil. Front Cell Dev Biol.

[28] Heidarian M., Griffith T.S., Badovinac V.P. (2023). Sepsis-induced changes in differentiation, maintenance, and function of memory CD8 T cell subsets. Front Immunol.

[29] Jubel J.M., Barbati Z.R., Burger C., Wirtz D.C., Schildberg F.A. (2020). The Role of PD-1 in Acute and Chronic Infection. Front Immunol.

[30] Knaus W.A., Draper E.A., Wagner D.P., Zimmerman J.E. (1985). APACHE II: a severity of disease classification system. Crit Care Med.

[31] Danahy D.B., Strother R.K., Badovinac V.P., Griffith T.S. (2016). Clinical and Experimental Sepsis Impairs CD8 T-Cell-Mediated Immunity. Crit Rev Immunol.

[32] Le Tulzo Y., Pangault C., Gacouin A., Guilloux V., Tribut O., Amiot L. (2002). Early circulating lymphocyte apoptosis in human septic shock is associated with poor outcome. Shock.

[33] Rodionov V.E., Avdalyan A.M., Konovalov D.M., Boriskin N.V., Tyurin I.N., Protsenko D.N. (2022). Features of the cell composition of inflammatory infiltrate in different phases of diffuse alveolar lung damage with COVID-19. Arkh Patol.

[34] Wherry E.J., Ha S.J., Kaech S.M., Haining W.N., Sarkar S., Kalia V. (2007). Molecular signature of CD8+ T-cell exhaustion during chronic viral infection. Immunity.

[35] Hotchkiss R.S., Monneret G., Payen D. (2013). Sepsis-induced immunosuppression: from cellular dysfunctions to immunotherapy. Nat Rev Immunol.

[36] Yan L., Chen Y., Yang Y., Han Y., Tong C. (2024). Heat shock protein 90α reduces CD8+ T-cell exhaustion in acute lung injury induced by lipopolysaccharide. Cell Death Discov.

