# Supplementary figures and images for: Comprehensive analysis of lymphocyte subsets and transcriptome profiles in sepsis-induced acute respiratory distress syndrome: a prospective, observational study
Source: Clinics (Sao Paulo). 2025 Aug 22;80:100754. doi: 10.1016/j.clinsp.2025.100754 (PMC12398266; doi:10.1016/j.clinsp.2025.100754)

A

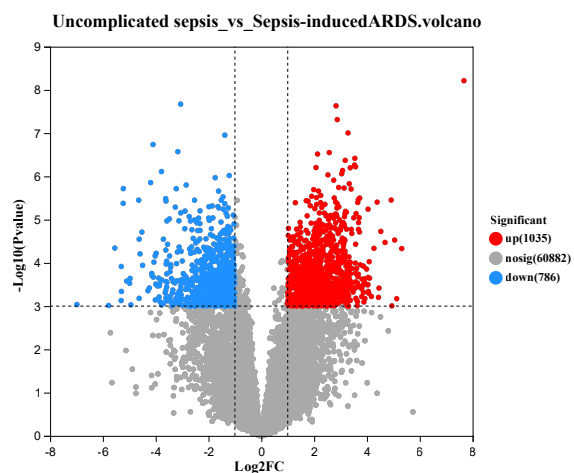

B

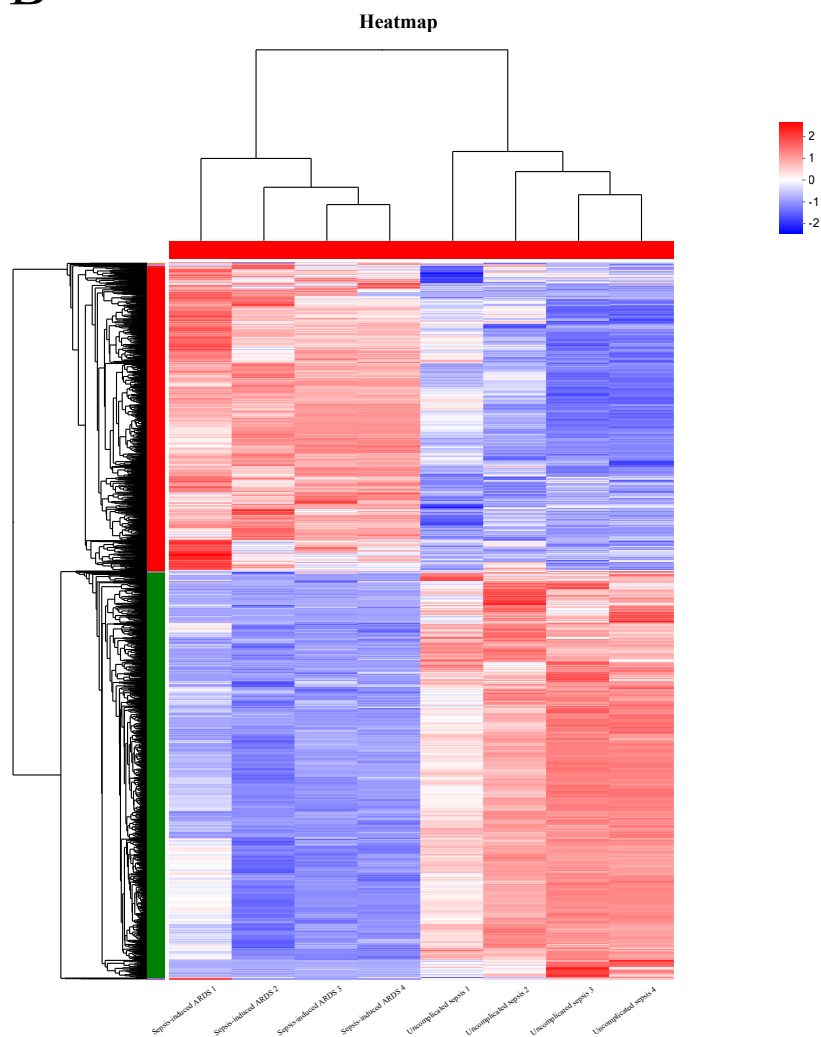

C

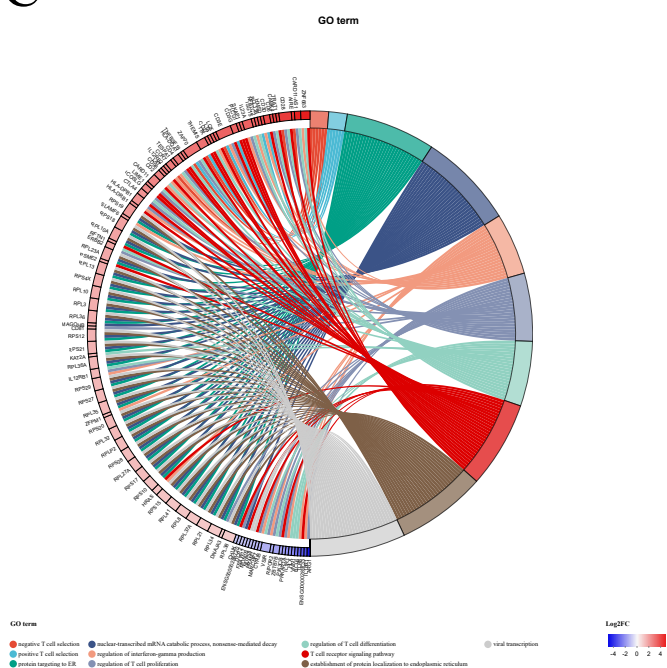

D

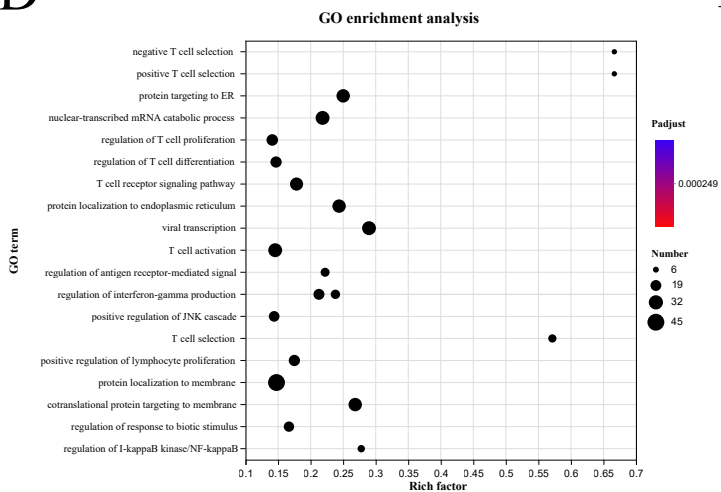

E

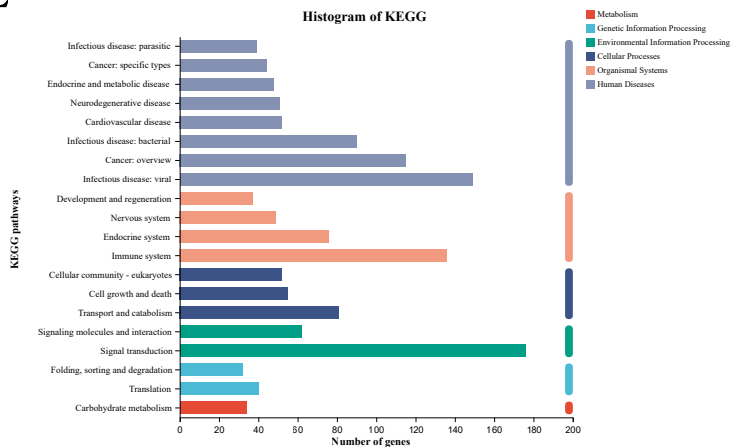

Supplement: Supplementary file 1 [file mmc1.pdf]
